# Audiovisual characteristics, patterns, and facial-expression responses in an under-viaduct green space

**DOI:** 10.3389/fpsyg.2026.1970940

**Published:** 2026-10-21

**Authors:** Yuting Yin, Feng Wang, Chengci Li, Dongbo Ma

**Affiliations:** 1 School of Design, East China Normal University, Shanghai, China; 2 Shanghai Tongji Urban Planning and Design Institute, Co. Ltd., Shanghai, China

**Keywords:** audiovisual patterns, facial-expression analysis, multisensory perception, semantic segmentation, under-viaduct green spaces (UVGSs)

## Abstract

**Introduction:**

Under-viaduct green spaces (UVGSs) combine natural landscape elements with persistent transport-related visual and acoustic cues, yet their audiovisual characteristics and experiential implications remain insufficiently understood.

**Methods:**

This exploratory study examined a UVGS in Shanghai using three recorded audiovisual scenarios representing different landscape and traffic conditions. Forty-nine participants viewed the stimuli in a controlled laboratory setting. Visual features were quantified through semantic segmentation, acoustic conditions through field sound-level measurements and 1-s sound-source prominence coding, and facial-expression responses through DeepFace-derived scores sampled at 1-s intervals. Hierarchical clustering was used to identify recurring audiovisual patterns.

**Results:**

The three scenarios differed clearly in visual composition, overall sound level, and sound-source composition, but exploratory component-wise Friedman tests showed no significant scenario-level differences in any of the seven relative DeepFace output components after Holm adjustment. Six audiovisual patterns were identified, with descriptive variation in facial-expression scores across the patterns. Because pattern occurrence was strongly associated with scenario and temporal position, these differences were interpreted descriptively rather than as independent pattern effects.

**Discussion:**

These scores represent relative facial-expression outputs rather than direct measures of subjective emotional states. Overall, the findings show that audiovisual experience in UVGSs is better understood by considering visual context, sound-source composition, and their co-occurrence rather than isolated environmental indicators.

## 1 Introduction

Under-viaduct green spaces (UVGSs) are distinctive urban green environments in which natural features are experienced in direct proximity to active transport infrastructure (Ma et al., 2026). As a result, vegetation and other natural cues coexist with traffic-related visual and acoustic information, creating audiovisual conditions whose emotional implications remain poorly understood. Previous research on spaces beneath transport infrastructure has focused primarily on reuse, activity functions, and environmental preferences (Mohamed Anuar and Ahmad, 2018; Nasr et al., 2024), with limited attention to how visual and acoustic features combine within UVGSs and how these combinations relate to emotional responses. This exploratory study integrates semantic segmentation, acoustic characterization, and facial-expression analysis to compare three audiovisual scenarios, identify recurring audiovisual patterns, and describe variation in DeepFace-derived facial-expression scores across these patterns. By moving beyond isolated visual or acoustic indicators, the study provides a more differentiated basis for examining audiovisual experience in green spaces embedded within transport infrastructure.

### 1.1 Restorative potential of under-viaduct green spaces under transport constraints

In high-density cities, the provision of conventional parks is increasingly constrained by limited and fragmented land resources, while visits to larger or more distant green spaces may be difficult to integrate into everyday routines (Peschardt et al., 2012). This has increased the importance of small, accessible green spaces embedded in everyday activity patterns. Spaces beneath elevated roads and railways have often been treated as residual or underutilized areas created by transport infrastructure. However, their central locations and close connections to surrounding neighborhoods and movement networks provide opportunities for public-space reuse (Kunimura et al., 2009), and recent studies have increasingly explored their potential for landscape and public-space transformation (Yang et al., 2024). When supplemented with vegetation, pedestrian facilities, and recreational functions, such spaces can expand opportunities for nearby public use and everyday contact with nature (Trancik, 1991).

The introduction of natural elements gives UVGSs potential relevance to psychological restoration (Kaplan and Kaplan, 1989; Nordh et al., 2009; Zhang et al., 2023). Attention Restoration Theory suggests that natural environments can replenish directed attention (Kaplan, 1995), whereas Stress Reduction Theory emphasizes their capacity to reduce stress and support affective recovery (Ulrich et al., 1991). Consistent with these perspectives, even brief exposure to urban nature has been associated with reduced negative affect and improvements in mood and emotional well-being (Yao et al., 2024). These findings establish the relevance of emotional experience to the present study, but they do not imply that restoration follows automatically from the presence of vegetation. Rather, the restorative potential of green spaces depends on the broader environmental conditions in which natural features are experienced (Ulrich et al., 1991; Hartig et al., 2003; Tyrväinen et al., 2014).

The environmental conditions of UVGSs differ substantially from those of conventional urban parks. Many parks achieve some separation from intensive traffic through distance, enclosure, vegetation belts, land-use transitions, or internal circulation systems (Van Renterghem and Botteldooren, 2016; Tashakkor et al., 2020). UVGSs, by contrast, are located directly beneath or alongside active transport infrastructure and often lack comparable visual and acoustic buffers. Bridge decks, piers, roads, railways, vehicles, and other transport-related elements remain visible within or immediately adjacent to these spaces, while traffic noise enters with limited attenuation. Natural features are therefore experienced together with persistent infrastructural cues rather than within a predominantly natural setting. Evidence from traffic-exposed green spaces further indicates that road noise can reduce perceived restorativeness, recreational suitability, and stress recovery even where vegetation is present (Gidlöf-Gunnarsson and Öhrström, 2007; Schaupp et al., 2025).

UVGSs may therefore be understood as restorative environments under persistent transport constraints. Their potential experiential value cannot be inferred from the presence or quantity of greenery alone, because natural features are encountered together with visual and acoustic information from surrounding infrastructure. What remains unclear is how these natural and transport-related cues combine within UVGSs and whether different combinations of these cues are associated with different emotional responses. Addressing this question requires attention to audiovisual interaction rather than to visual or acoustic conditions in isolation.

### 1.2 Audiovisual interaction and emotional responses in under-viaduct green spaces

In complex environments such as UVGSs, visual and auditory cues are not perceived independently. Cross-modal integration allows visual context to shape expectations about the identity, location, and appropriateness of sounds (Özcan and van Egmond, 2009; Spence, 2011), while acoustic information can influence how the surrounding landscape is interpreted and evaluated (Anderson et al., 1983; Carles et al., 1999; Ren et al., 2016; Li et al., 2026). The same sound may therefore be judged differently in different visual settings (Viollon et al., 2002), while changes in sound type or quality can alter perceived tranquility, pleasantness, naturalness, and restorative value (Pheasant et al., 2010; Yan et al., 2025). Emotional responses to environmental settings are consequently shaped by the combined visual and acoustic context rather than by either modality alone (Gifford and Ng, 1982; Viollon et al., 2002; Jeon and Jo, 2020; Jiang et al., 2021).

One important aspect of this interaction is the compatibility between visual and auditory information. Audiovisual congruence describes whether what is seen and heard is perceptually consistent, but congruence does not necessarily imply a positive emotional outcome (Jo and Jeon, 2020). Natural scenes paired with natural sounds and transport scenes paired with traffic sounds may both be congruent while producing different affective evaluations (Carles et al., 1992). Findings for incongruent combinations are less consistent: visible natural elements may reduce noise annoyance in some settings (Van Renterghem and Botteldooren, 2016; Pasanen et al., 2025), whereas anthropogenic sounds may diminish the appraisal of natural landscapes in others (Aldoh et al., 2025). Emotional responses therefore depend not only on congruence, but also on the content, prominence, and compatibility of the cues involved (Pheasant et al., 2008; Benfield et al., 2010; Fukunaga et al., 2026).

This issue is particularly relevant to UVGSs, where natural and transport-related cues coexist and vary within the same setting. Vegetation and water may occur with bird vocalizations or flowing water, but they may also be accompanied by traffic sounds; roads, vehicles, and bridge structures may likewise coincide with road or railway noise. UVGSs therefore cannot be reduced to a single form of audiovisual congruence or incongruity.

Existing audiovisual studies have commonly relied on predefined combinations presented through still images, videos, virtual environments, or controlled sound-image pairings (Hetherington et al., 1993; Li and Lau, 2020; Latini et al., 2024). Although such designs are effective for comparing controlled conditions, they provide less insight into how visual and acoustic features coexist and vary within complex real-world settings. It therefore remains unclear how audiovisual conditions are structured within UVGSs, whether scenarios with different visual and acoustic characteristics correspond to different emotional responses, and whether recurring combinations within those scenarios are accompanied by differences in observable facial expression. Addressing these questions requires both comparison of overall audiovisual conditions and finer-grained examination of recurring audiovisual patterns.

### 1.3 Study aim and research questions

Existing research provides limited insight into audiovisual experience in UVGSs. Studies of spaces beneath transport infrastructure have rarely examined how visual and acoustic conditions jointly relate to emotional responses, while audiovisual research has largely relied on predefined and comparatively controlled combinations of scenes and sounds (Lak et al., 2019; Li et al., 2022; Parmar and Jana, 2024). Consequently, how visual and acoustic features combine and vary within UVGSs remains insufficiently understood.

This exploratory study examines the audiovisual conditions of a UVGS and the facial-expression responses observed during exposure. DeepFace-derived scores are treated as measures of observable facial-expression responses rather than as direct measures of subjective emotional states.

Accordingly, the study addresses three research questions:

RQ1. How do the three audiovisual scenarios differ in their visual and acoustic characteristics and facial-expression scores?
RQ2. How do visual and acoustic features vary within the scenarios, and what recurring audiovisual patterns can be identified?
RQ3. What variation in facial-expression scores is observed across the identified audiovisual patterns?

Together, these questions provide a basis for examining audiovisual experience in UVGSs at both overall scenario and pattern levels.

## 2 Materials and methods

### 2.1 Research site

Jing’an Zhonghuan Park in Shanghai was selected because it combines landscaped green areas with direct and persistent exposure to major transport infrastructure, providing contrasting audiovisual conditions within a single UVGS. The park is located beneath the interchange of the Middle Ring Road and the North–South Elevated Road at Gonghexin Road in Jing’an District. It is divided into four green parcels by Wenshui Road and Gonghexin Road and covers approximately 78,000 m^2^. The site was redeveloped between 2016 and 2018 and was formally included in the Shanghai Urban Park Register in 2019 (Shanghai Landscaping and City Appearance Administrative Bureau, 2019).

The park contains both vegetation-dominated recreational areas and spaces in which viaduct structures, surface roads, moving vehicles, and other transport facilities are visually prominent. Traffic noise is also continuously present, with clear differences between peak and off-peak periods. These natural and transport-related visual and acoustic features made the site suitable for recording contrasting audiovisual scenarios while retaining the context of the same under-viaduct green space. These conditions also provided sufficient within-site variation for examining changes within scenarios and identifying recurring audiovisual patterns. The location and spatial setting of Jing’an Zhonghuan Park are shown in Figure 1.

**Figure 1:**
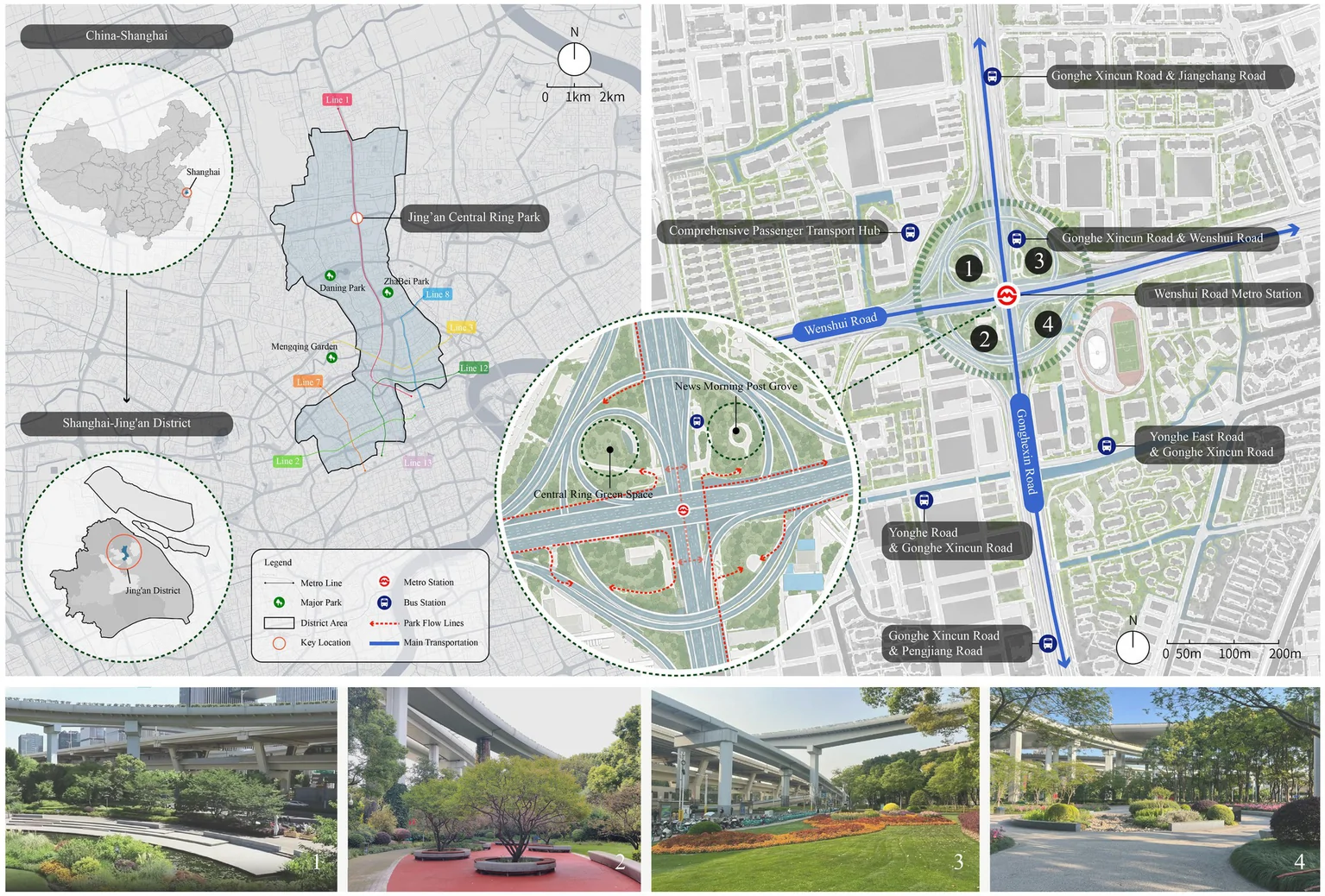
Location and spatial setting of Jing’an Zhonghuan Park.

### 2.2 Audiovisual stimuli

Recorded audiovisual stimuli were used to present the environmental conditions under standardized laboratory conditions while retaining the principal visual and acoustic characteristics of the study site. This approach reduced variation in incidental environmental and social factors that might otherwise influence facial expressions during on-site assessment (Li et al., 2021). Recorded environmental stimuli have been widely used to examine perceptual and emotional responses under controlled conditions (Hetherington et al., 1993; Li et al., 2023).

Field investigation identified two contrasting visual settings within the park. Interior areas were characterized mainly by planted landscapes, whereas areas closer to the transport infrastructure provided direct views of roads, viaduct structures, vehicles, and related facilities. Acoustic conditions also differed between off-peak and peak traffic periods, as indicated by field-measured $L_{\mathrm{Aeq}}$ values. These visual settings and traffic conditions provided the basis for selecting the three audiovisual scenarios shown in Figure 2.

**Figure 2:**
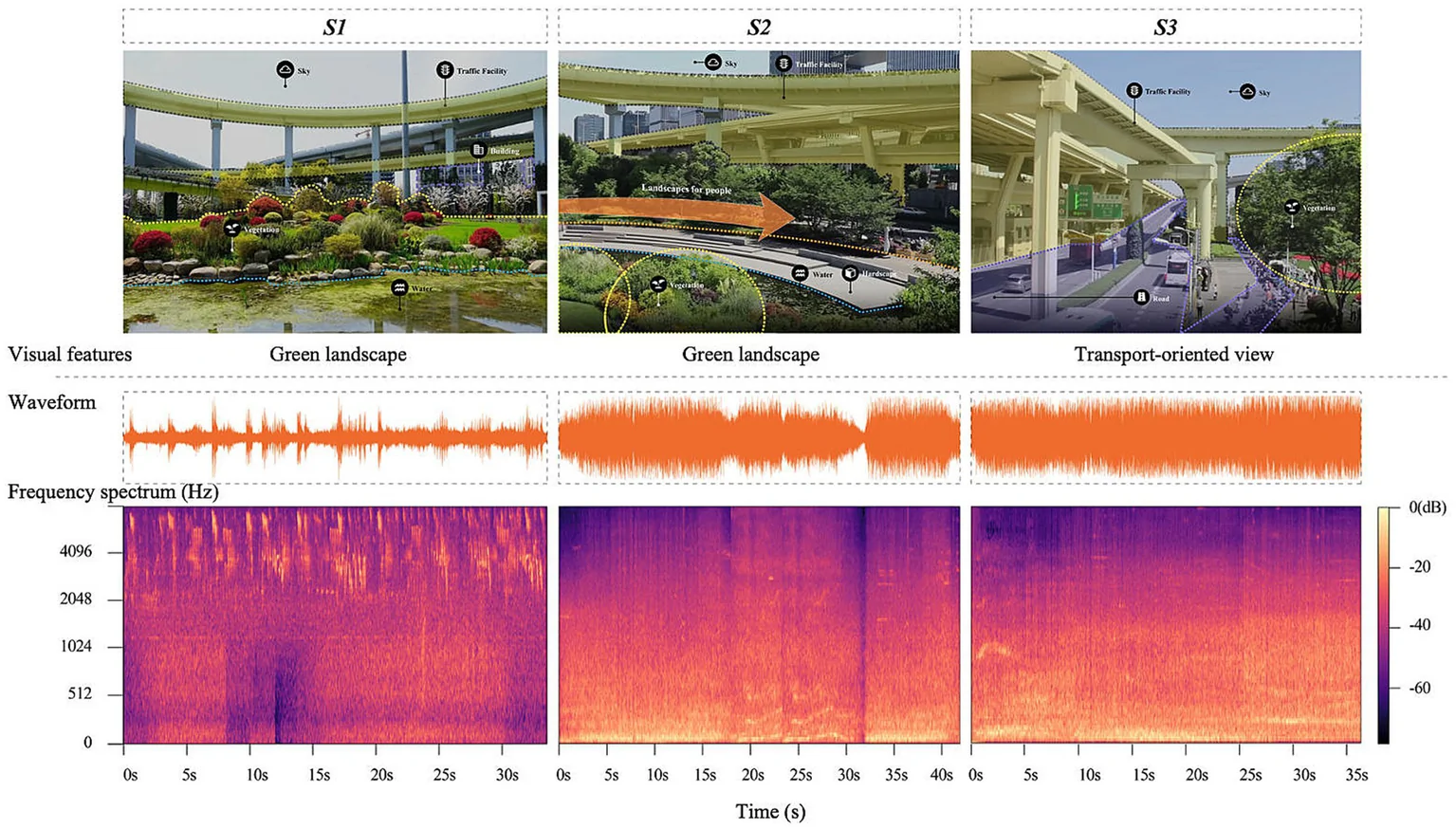
Visual scenes, audio waveforms, and spectrograms of the three scenarios.

S1 represented a green landscape recorded during off-peak traffic conditions, S2 a green landscape during peak traffic conditions, and S3 a transport-oriented view during peak traffic conditions. The three scenarios were selected to represent contrasting audiovisual conditions observed within the same UVGS rather than to isolate independent visual or acoustic effects. The fourth possible combination, a transport-oriented view during off-peak traffic conditions, was not included; the study therefore used representative field conditions rather than a fully crossed factorial design. The three scenarios were compiled into a single continuous stimulus video and presented in a fixed order from S1 to S3. An initial 10-s immersion period preceded the analyzed portion of S1, and smooth transition segments were inserted between scenarios to reduce abrupt perceptual disruption. The immersion period and transition segments were excluded from subsequent analyses. The retained durations were 33 s for S1, 41 s for S2, and 36 s for S3, yielding a total of 110 s of retained stimulus material.

### 2.3 Participants and procedure

Forty-nine undergraduate students participated in the experiment, including 35 females and 14 males. Ages ranged from 20 to 28 years (M = 22, SD = 1.43). All participants reported normal hearing and normal or corrected-to-normal vision, completed the experiment, and were included in the analyses. Data were collected over one week in June 2025. Ethical approval was obtained prior to data collection, and informed consent was obtained from all participants. Participant privacy and personal data were protected throughout the study.

The experiment was conducted in a laboratory area enclosed on three sides by white walls to reduce external visual and auditory interference. The stimulus was projected onto a 69 × 39 cm area, with participants seated approximately 1.5 m from the screen. The same over-ear noise-canceling headphones (Salar E28) and playback device were used for all participants at a fixed volume of 60%; no ear-level sound-pressure calibration was performed. Supplementary white lighting and fixed seating were used to maintain consistent facial illumination and minimize backlighting and facial shadows. The experimental setup is shown in Figure 3a.

**Figure 3:**
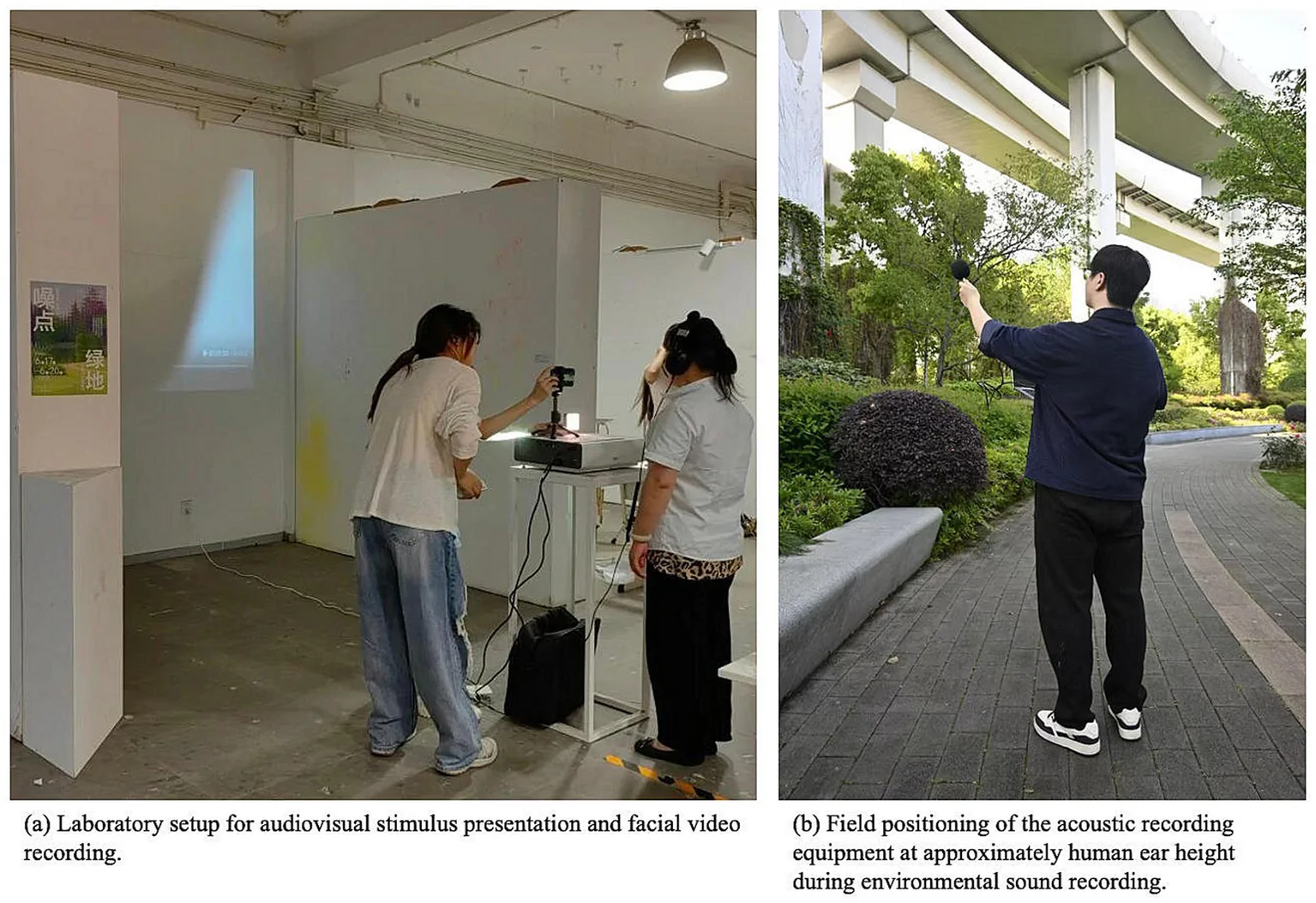
Experimental and field data collection setups. **(a)** Laboratory setup for audiovisual stimulus presentation and facial video recording. **(b)** Field positioning of the acoustic recording equipment at approximately human ear height during environmental sound recording.

Facial video was recorded continuously using an iPhone 16 Pro at 1080p and 60 fps. The device was positioned approximately 20 cm in front of the participant at a height of 120 cm and tilted upward by 15°, providing a clear frontal view without substantially obstructing the stimulus. Facial recording was synchronized with stimulus playback.

Before the experiment, participants received standardized oral and written instructions. Each participant viewed the continuous stimulus once, with S1, S2, and S3 presented in fixed order, and was instructed to attend naturally to the audiovisual content while avoiding unnecessary head and body movements.

### 2.4 Data extraction and characterization

Three types of data were obtained from the audiovisual stimuli and synchronized facial recordings: visual features, acoustic features, and facial-expression scores. Visual composition and facial-expression scores were sampled at fixed 1-s intervals, while sound-source prominence was coded at the same temporal resolution; field measurements were used separately to characterize the overall sound level of each scenario. The 1-s interval was selected as a common temporal unit because it was compatible with the temporal resolution of sound-source coding and enabled consistent frame-based extraction and alignment of the visual and facial data. It therefore provided a practical basis for describing within-scenario variation across the three data streams, rather than representing a discrete psychological response window or implying that perceptual or affective responses occurred independently within each second. The aligned data were subsequently used in the scenario- and pattern-level analyses.

#### 2.4.1 Visual features

One frame was extracted at each fixed 1-s time point from the stimulus video and analyzed using semantic segmentation models trained on the ADE20K and Cityscapes datasets. The ADE20K-based model was used mainly to identify landscape and built-environment features, including vegetation, water, sky, terrain, buildings, and hardscape, whereas the Cityscapes-based model provided more detailed recognition of transport-related features, including roads, vehicles, persons, and traffic facilities (Kim et al., 2022).

Because the two datasets differ in class definitions and level of detail, their outputs were mapped to a common classification comprising ten visual categories: vegetation, water, sky, terrain, building, hardscape, road, vehicle, traffic facility, and person. When the two model outputs overlapped or conflicted, Cityscapes classifications were given priority for transport-specific categories, including roads, vehicles, persons, and traffic facilities, whereas ADE20K classifications were retained for landscape and general built-environment categories. Remaining ambiguous regions, inaccurate boundaries, and apparent classification errors were reviewed against the original frames and corrected where necessary.

After model integration and verification, each pixel was assigned to one mutually exclusive category. The proportion of each visual feature was calculated by dividing the number of pixels assigned to that category by the total number of pixels in the frame. The resulting ten proportions represented the visual composition at each 1-s time point.

#### 2.4.2 Acoustic features

Acoustic conditions were characterized at two levels: overall sound level for each recording condition and sound-source composition at 1-s intervals. Field measurements were conducted concurrently with the audiovisual recordings using two AWA14423 measurement microphones (20–16,000 Hz; approximately 40 mV/Pa) connected to an AWA6290L + multichannel signal analyzer. Each microphone was fitted with an 80-mm windshield and mounted approximately 1.6 m above ground, corresponding to human ear height at the recording location. Figure 3b illustrates the field procedure used to position a measurement microphone at approximately this height.

For each recording condition, the A-weighted equivalent continuous sound pressure level ( $L_{\mathrm{Aeq}}$ ) was measured continuously for 10 min to characterize the overall acoustic environment of the three scenarios. Because the laboratory stimuli were reproduced through headphones at a fixed device volume without ear-level sound-pressure calibration, these field measurements describe the recorded environmental conditions rather than participants’ individual auditory exposure.

Sound-source composition was analyzed from the stimulus audio tracks at 1-s intervals using the pretrained YAMNet model based on AudioSet (Gemmeke et al., 2017). Detected events were grouped into five categories: bird vocalizations, flowing water, tire-road interaction, engine noise, and railway noise. Automated classifications were checked against the corresponding spectrograms and audible content in Sonic Visualiser (Cannam et al., 2010), and classification errors were corrected manually. The complete dataset was reviewed twice by the same researcher for consistency.

After verification, each sound source was assigned a prominence score from 0 to 3: 0 indicated absence or unreliable identification, 1 a weak background source, 2 a clearly audible but non-dominant source, and 3 a highly prominent or dominant source. Multiple sources within the same interval were scored independently. The five prominence scores represented the sound-source composition of each 1-s interval.

#### 2.4.3 Facial-expression classification

The facial recordings were sampled at fixed 1-s time points to match the temporal resolution of the visual and sound-source analyses. One frame was extracted at each time point and processed using DeepFace (version 0.0.93; detector backend: OpenCV). A frame was considered valid when DeepFace returned a facial-expression output; no additional user-defined detection-confidence threshold was applied. Invalid outputs occurred only during the initial immersion or transition periods and did not affect the retained data.

After exclusion of these periods, 110 s of stimulus material remained: 33 s for S1, 41 s for S2, and 36 s for S3. All 49 participants had valid DeepFace outputs at all retained time points, yielding 5,390 participant-by-time-point observations. The 1-s resolution served as a common sampling and synchronization unit across the multimodal data and should not be interpreted as a discrete psychological response window or as evidence of instantaneous facial responses within the same second.

For each valid image, DeepFace generated percentage scores for seven categories: angry, disgust, fear, happy, sad, surprise, and neutral. The complete seven-category output was retained. Because the scores summed to 100%, they formed a seven-part composition and were not statistically independent. In subsequent analyses, these values are referred to as facial-expression scores and are interpreted as measures of observable facial-expression responses rather than direct measures of subjective emotional states. Any interpretation in relation to underlying emotional responses is therefore inferential.

### 2.5 Data analysis

Analyses were organized around the three research questions. Normality of participant-level scenario means was assessed using Shapiro–Wilk tests, and nonparametric repeated-measures tests were used where appropriate.

For RQ1, visual composition, sound level, sound-source composition, and participant-level facial-expression scores were compared across S1, S2, and S3. Because the seven DeepFace scores formed a composition summing to 100%, separate Friedman tests were treated as exploratory component-wise comparisons rather than tests of independent outcomes. Holm adjustment was applied across the seven tests.

For RQ2, visual and acoustic features were examined across successive 1-s intervals. Recurring audiovisual patterns were identified by hierarchical clustering of the standardized ten visual variables and five sound-source prominence scores using Ward’s linkage and Euclidean distance. The number of clusters was selected based on statistical indices, cluster size, and interpretability.

For RQ3, facial-expression scores across the six audiovisual patterns were summarized descriptively. For each retained 1-s interval, each of the seven DeepFace component scores was first averaged across the 49 participants; the resulting interval-level mean scores were then grouped by audiovisual pattern and averaged across the intervals assigned to each pattern. No inferential comparison was conducted across all six patterns because adjacent intervals were temporally dependent and pattern occurrence was strongly associated with scenario and temporal position. As an exploratory sensitivity analysis within S2, mean relative scores were calculated for each participant across the intervals assigned to P2, P5, and P6 and compared using component-wise Friedman tests with Holm adjustment. P1 was excluded because it occurred in only two intervals. No equivalent analysis was conducted within S3 because P3, P4, and P5 occurred in successive non-overlapping temporal blocks and could not be separated from temporal position.

## 3 Results

Before the main analyses, data completeness and distributional characteristics were examined. After excluding the initial immersion and transition periods, 110 s of stimulus material remained: 33 s for S1, 41 s for S2, and 36 s for S3. With 49 participants sampled at 1-s intervals, this yielded 5,390 participant-by-time-point observations, all of which had valid DeepFace outputs (100%).

Normality was assessed for the participant-level mean score of each facial-expression category within each scenario. Shapiro–Wilk tests indicated significant departures from normality for all 21 distributions (all *p* < 0.05). Nonparametric repeated-measures methods were therefore used for the subsequent scenario-level comparisons.

### 3.1 Audiovisual characteristics across scenarios

#### 3.1.1 Visual characteristics

The semantic segmentation results showed distinct visual compositions among the three scenarios (Figure 4). For descriptive comparison, vegetation, water, sky, and terrain were grouped as natural features, whereas building, hardscape, roads, vehicles, and traffic facilities were grouped as built and transport-related features; the person category was reported separately.

**Figure 4:**
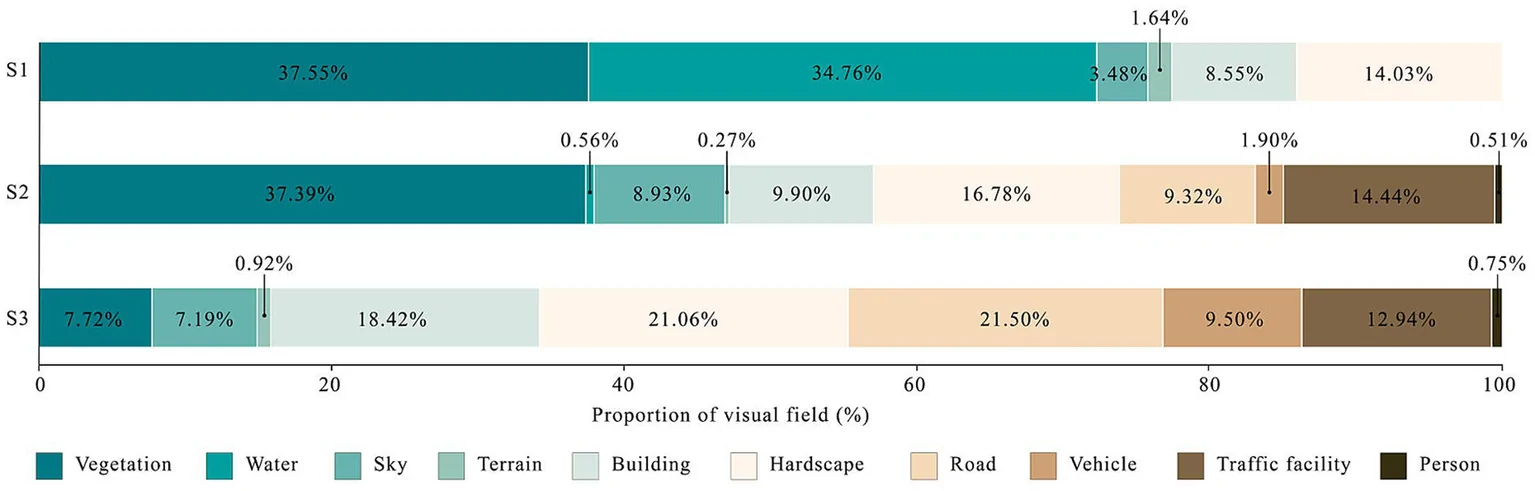
Mean proportions of visual features across the three audiovisual scenarios.

S1 was predominantly composed of natural features, which accounted for 77.43%. Vegetation (37.55%) and water (34.76%) were the two largest individual categories, whereas built and transport-related features accounted for 22.58%. S2 showed a more mixed composition. Vegetation remained the largest individual category at 37.39%, while natural features collectively accounted for 47.15% and built and transport-related features for 52.34%; the person category accounted for the remaining 0.51%. In S3, built and transport-related features increased to 83.42%, while natural features decreased to 15.83%; the person category accounted for 0.75%. Road (21.50%) and hardscape (21.06%) were the two largest individual categories, followed by building (18.42%), whereas vegetation accounted for only 7.72%.

#### 3.1.2 Acoustic characteristics

Field measurements conducted concurrently with the audiovisual recordings showed clear differences in overall sound level among the three scenarios. The 10-min A-weighted equivalent continuous sound pressure level ( $L_{\mathrm{Aeq}}$ ) was 54.5 dB(A) for S1 during the off-peak period, compared with 74.2 dB(A) for S2 and 76.7 dB(A) for S3 during the peak period.

Sound-source composition also differed among the three scenarios (Figure 5). In S1, bird vocalizations were detected in 96.97% of the 1-s intervals and flowing water in 66.67%, with mean prominence scores of 2.03 (SD = 0.92) and 1.00 (SD = 0.83), respectively. Transport-related sounds occurred only intermittently and at low prominence levels. In S2, tire-road interaction was detected in every interval and had a mean prominence score of 2.39 (SD = 0.54). Engine noise occurred in 97.56% of the intervals, with a mean prominence score of 1.95 (SD = 0.80). Natural sounds were infrequent and generally weak. In S3, tire-road interaction was detected in every interval, with a mean prominence score of 1.83 (SD = 0.56). Engine noise occurred in 75.00% of the intervals, while railway noise occurred in 25.00%. Railway noise was specific to S3 and had a mean prominence score of 0.67 (SD = 1.20).

**Figure 5:**
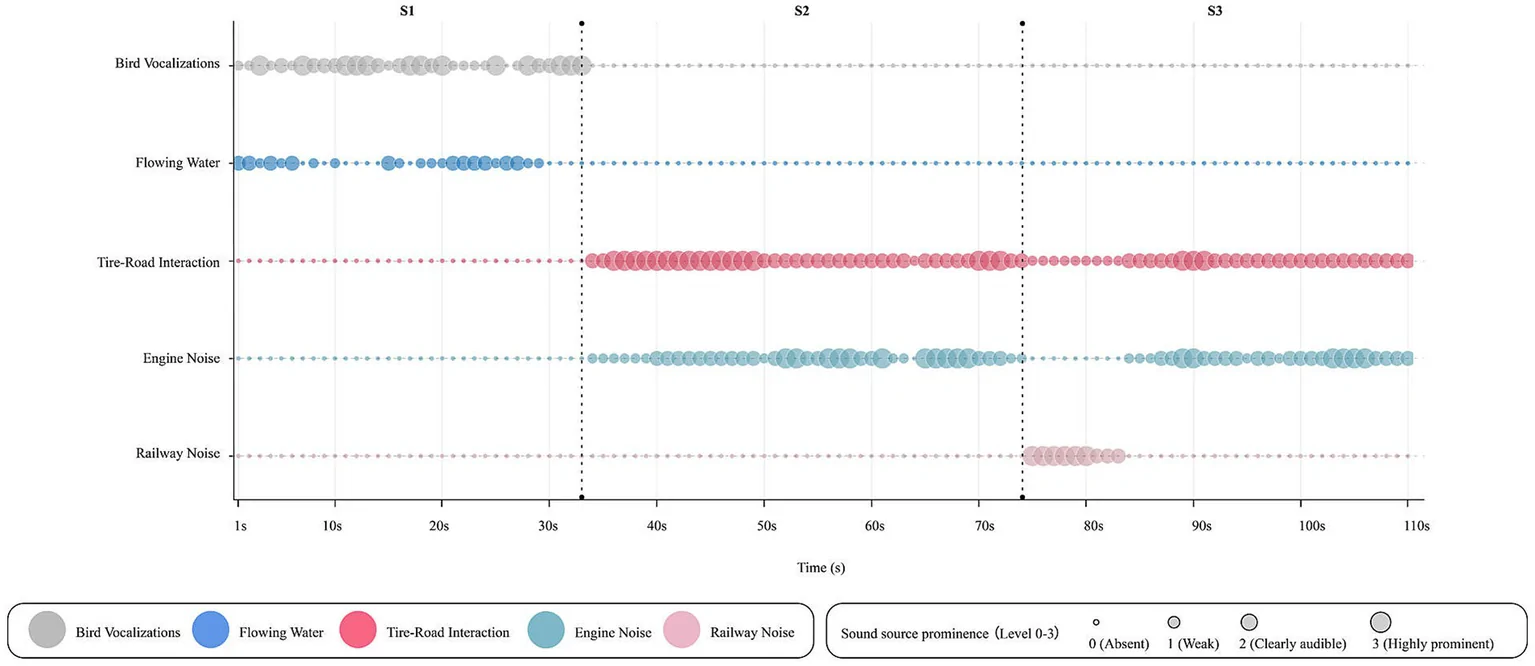
Sound-source prominence across successive 1-s intervals in the three scenarios. Marker size represents prominence on a four-level scale from 0 (absent) to 3 (highly prominent). Vertical dashed lines indicate the boundaries between S1, S2, and S3.

#### 3.1.3 Within-scenario variation in audiovisual features

Analysis at 1-s intervals showed variation in both visual composition and sound-source prominence within each scenario (Figure 6). Because the field measurements provided scenario-level $L_{\mathrm{Aeq}}$ values, within-scenario acoustic variation was characterized using the five sound-source prominence scores.

**Figure 6:**
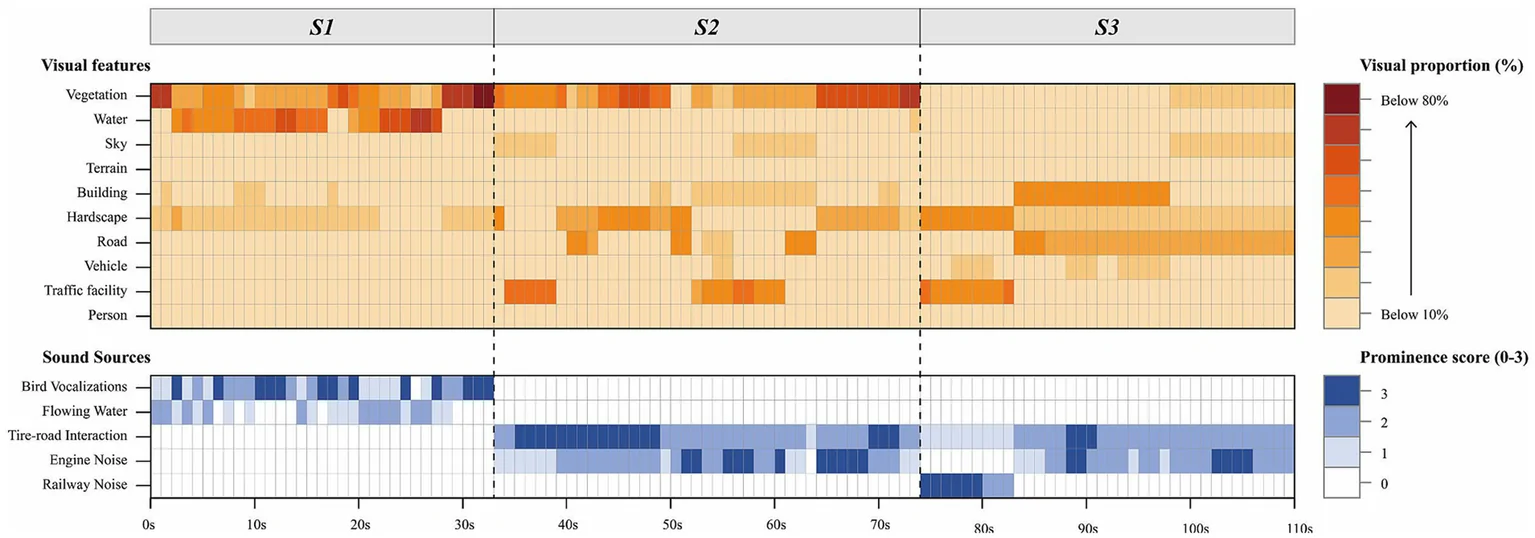
Variation in visual composition and sound-source prominence across the 110 retained 1-s intervals. The upper panel shows the proportional area of the ten visual features, and the lower panel shows prominence scores for the five sound sources. Vertical dashed lines indicate the boundaries between S1, S2, and S3.

In S1, vegetation ranged from 20 to 72% of the visual field (SD = 18 percentage points), while water showed the greatest variation among the visual features, ranging from 0 to 65% (SD = 22 percentage points). Bird vocalizations occurred in most intervals, whereas flowing water was detected less consistently. Both natural sound sources varied in prominence, while transport-related sounds were generally weak and intermittent.

In S2, vegetation ranged from 6 to 64% of the visual field (SD = 16 percentage points). Traffic facilities showed substantial variation, ranging from 0 to 47% (SD = 19 percentage points), while hardscape and road also reached higher proportions in several intervals. Tire-road interaction was detected throughout the scenario at prominence levels of 1–3, and engine noise also occurred across much of the segment. Natural sounds were generally absent or weak.

In S3, hardscape, road, vehicle, building, and traffic facilities varied across successive intervals. Traffic facilities ranged from 3 to 42% of the visual field (SD = 16 percentage points). Tire-road interaction was detected throughout the scenario at prominence levels of 1–3, whereas engine and railway noise occurred less consistently. Railway noise was concentrated in the first 6 s of S3, after which road-traffic sounds constituted the principal acoustic components.

### 3.2 Facial-expression scores across scenarios

#### 3.2.1 Distribution of relative facial-expression scores

Table 1 summarizes the participant-level mean relative facial-expression scores across the three scenarios. The neutral component had the highest mean relative score in all scenarios, ranging from 30.31 to 34.03%, while fear was the largest non-neutral component, ranging from 18.57 to 20.86%. Descriptively, S1 showed the highest mean relative happy (9.64%) and surprise (8.92%) scores and the lowest mean relative sad score (14.54%). The mean relative fear score was highest in S3 (20.86%), whereas the mean relative angry score was highest in S2 (15.92%). Disgust remained close to zero across all three scenarios, with mean relative scores ranging from 0.07 to 0.15%.

**Table 1:** Participant-level mean relative facial-expression scores across the three audiovisual scenarios.

Scenario	Statistics	Angry	Disgust	Fear	Happy	Sad	Surprise	Neutral
S1	Mean	15.72	0.07	18.57	9.64	14.54	8.92	32.55
	Std. Dev	18.93	0.21	17.00	15.50	13.15	16.14	25.72
S2	Mean	15.92	0.10	18.93	8.32	18.69	7.72	30.31
	Std. Dev	16.73	0.37	18.52	12.13	17.62	14.87	23.43
S3	Mean	14.18	0.15	20.86	7.53	16.84	6.43	34.03
	Std. Dev	14.25	0.51	21.53	10.41	15.04	12.70	24.00

Mean values are relative percentage scores, and SDs are expressed in percentage points. *n* = 49 participants. Scores were averaged within each participant and scenario using only the retained analytical intervals (33 s for S1, 41 s for S2, and 36 s for S3). The seven DeepFace output components sum to 100% and therefore form a compositional outcome. Category labels correspond to the DeepFace model outputs and should not be interpreted as statistically independent outcomes or as direct measures of subjective emotional states.

#### 3.2.2 Differences in relative facial-expression scores among scenarios

Separate exploratory Friedman tests were conducted for each of the seven components to examine differences in relative scores among S1, S2, and S3 at the participant level. None was statistically significant before adjustment: angry, χ^2^ (2) = 1.143, *p* = 0.565; disgust, χ^2^ (2) = 3.148, *p* = 0.207; fear, χ^2^ (2) = 0.776, *p* = 0.679; happy, χ^2^ (2) = 0.292, *p* = 0.864; sad, χ^2^ (2) = 4.571, *p* = 0.102; surprise, χ^2^ (2) = 3.167, *p* = 0.205; and neutral, χ^2^ (2) = 2.287, *p* = 0.319. After Holm adjustment across the seven tests, all remained non-significant (all adjusted *p* ≥ 0.712). These analyses were interpreted as exploratory marginal comparisons of relative component scores rather than as tests of independent outcomes or as a global test of the seven-part composition.

#### 3.2.3 Temporal variation in facial-expression scores within scenarios

Facial-expression scores were averaged across participants at each 1-s interval to describe temporal variation within the three scenarios (Figure 7). Because the seven scores summed to 100% at each interval, they represented a relative composition rather than independent measures. The neutral component remained the largest at most intervals in all three scenarios.

**Figure 7:**
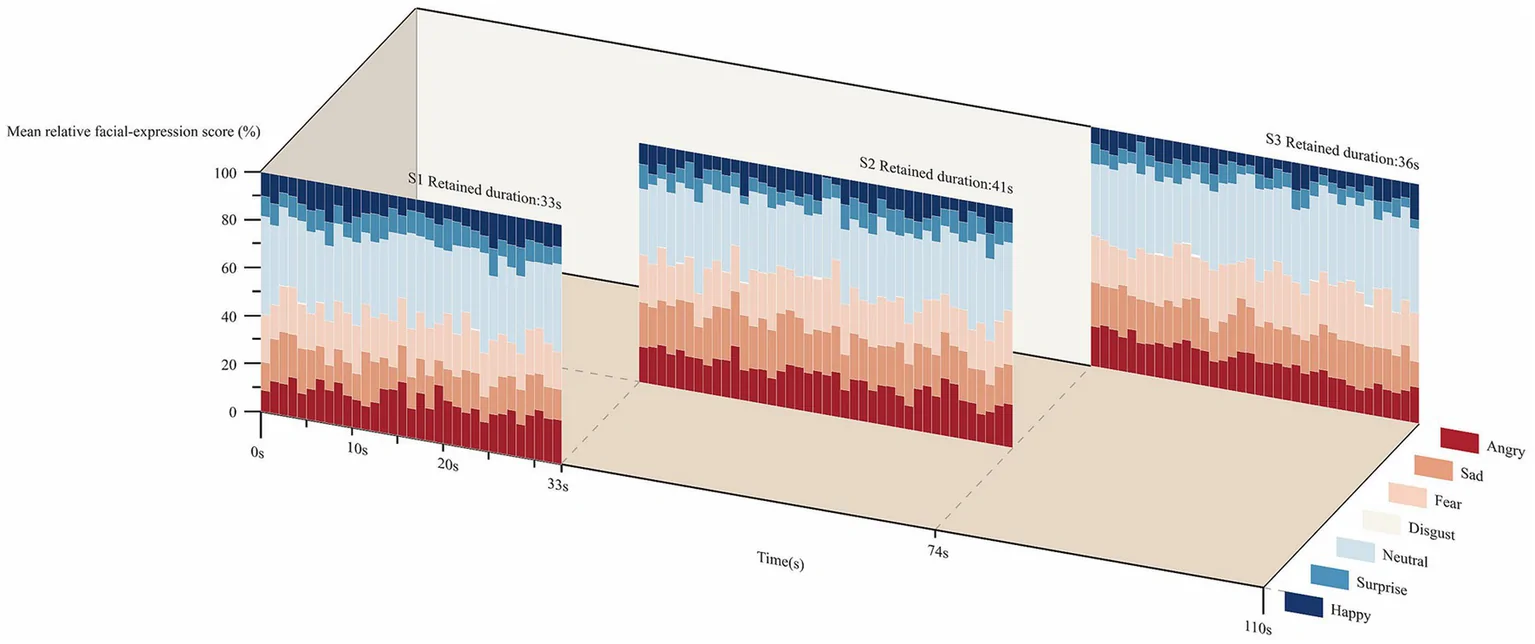
Mean relative facial-expression scores across successive 1-s intervals in S1, S2, and S3. Values represent interval-level mean relative facial-expression scores averaged across the 49 participants. The seven DeepFace output components sum to 100% at each interval and therefore constitute a relative composition rather than statistically independent outcomes.

The neutral score varied over spans of 15.71, 13.89, and 19.07 percentage points in S1, S2, and S3, respectively. The relative fear score also varied across intervals, with SDs of 2.27, 3.16, and 3.25 percentage points, respectively. The remaining components likewise varied over time, but no scenario showed a consistent increasing or decreasing pattern. These interval-level variations were treated descriptively and were not interpreted as evidence of instantaneous changes in subjective emotional state attributable to audiovisual characteristics within the same 1-s interval.

### 3.3 Audiovisual patterns and facial-expression scores

#### 3.3.1 Identification of audiovisual patterns

Hierarchical clustering was applied to the standardized visual and sound-source features of the 110 retained 1-s intervals and yielded six audiovisual patterns. The six-cluster solution had a silhouette coefficient of 0.512, a Calinski-Harabasz index of 62.514, and a Davies-Bouldin index of 0.843. Although the eight-cluster solution produced a slightly higher silhouette coefficient, inspection of the resulting profiles indicated that several clusters represented subdivisions of closely similar patterns. The six-cluster solution was therefore retained as a more parsimonious and interpretable representation of the data.

Figure 8 shows the standardized visual and sound-source profiles of the six patterns. Positive and negative z-scores indicate values above and below the overall mean, respectively, across the 110 retained intervals. Pattern labels were assigned descriptively based on the visual features and sound sources that most clearly distinguished each cluster.

**Figure 8:**
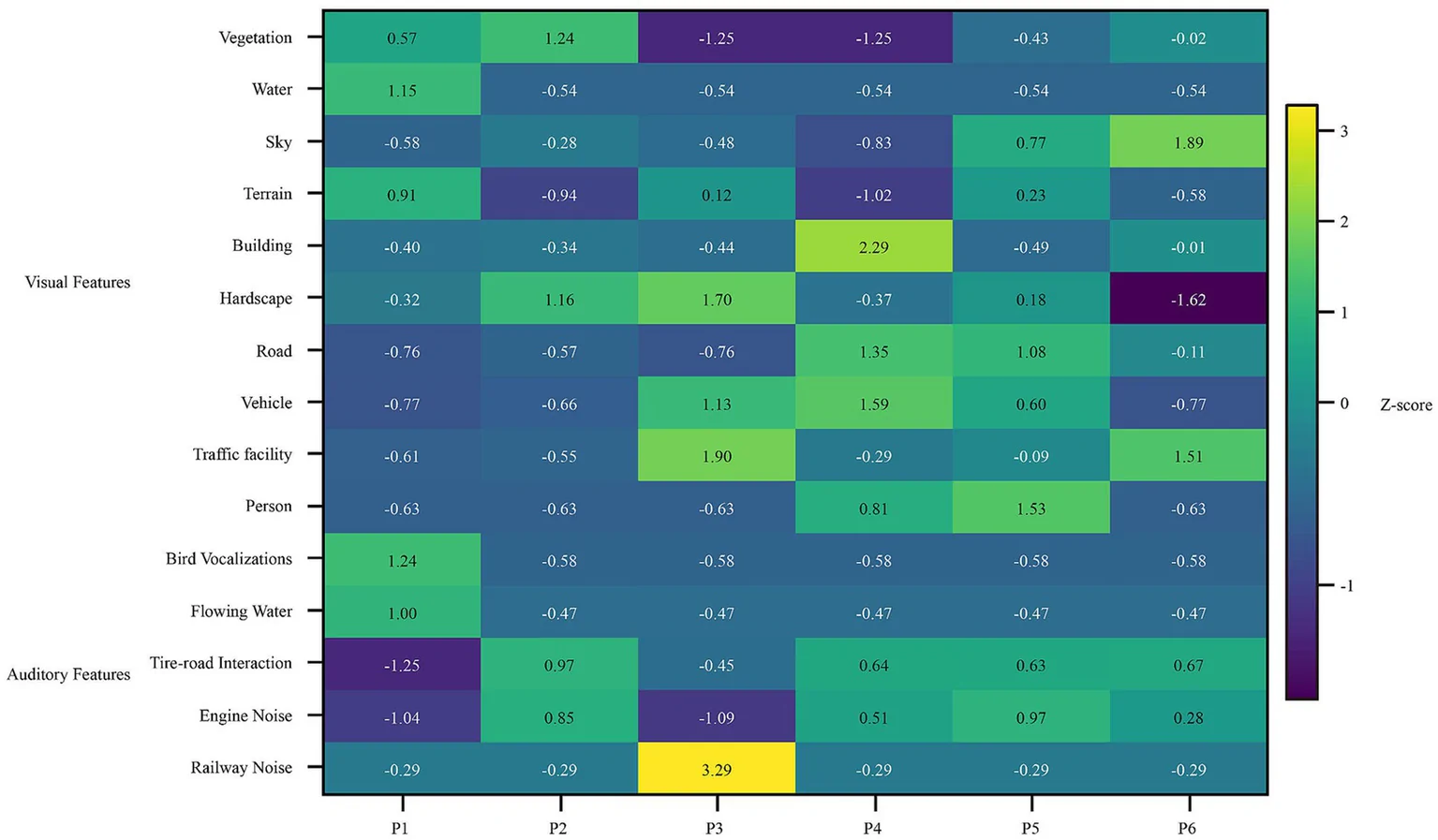
Standardized visual features and sound-source profiles of the six audiovisual patterns. Values are cluster means of variables standardized across the 110 retained 1-s intervals. A z-score of 0 represents the overall mean; positive and negative values indicate values above and below the overall mean, respectively.

P1 was characterized by vegetation, water, and terrain together with bird vocalizations and flowing water and was labeled Natural landscape. P2 combined vegetation and hardscape with tire-road interaction and engine noise and was labeled Green landscape with traffic sounds. P3 was distinguished by traffic facilities, hardscape, vehicles, and railway noise and was labeled Railway infrastructure. P4 combined buildings, roads, and vehicles with tire-road interaction and engine noise and was labeled Built road corridor, whereas P5 combined roads, sky, vehicles, and person presence with similar traffic sounds and was labeled Open road corridor. P6 combined traffic facilities, sky, and vegetation with tire-road interaction and engine noise and was labeled Traffic corridor with vegetation. Frequencies and characteristic features of the six patterns are summarized in Table 2.

**Table 2:** Characteristics and frequencies of the six audiovisual patterns.

Pattern	Pattern label	Characteristic visual features	Characteristic sound sources	*n* (%)
P1	Natural landscape	Vegetation, water, terrain	Bird vocalizations, flowing water	35 (31.82)
P2	Green landscape with traffic sounds	Vegetation, hardscape	Tire-road interaction, engine noise	16 (14.55)
P3	Railway infrastructure	Traffic facility, hardscape, vehicle	Railway noise	9 (8.18)
P4	Built road corridor	Building, road, vehicle	Tire-road interaction, engine noise	15 (13.64)
P5	Open road corridor	Road, sky, vehicle, person	Engine noise, tire-road interaction	22 (20.00)
P6	Traffic corridor with vegetation	Traffic facility, sky, vegetation	Tire-road interaction, engine noise	13 (11.82)

*n* indicates the number of 1-s intervals assigned to each pattern. Characteristic features summarize the variables that contributed most to the descriptive interpretation of each cluster based on the standardized cluster profiles and contrasts among patterns.

#### 3.3.2 Distribution of audiovisual patterns across scenarios

Table 3 summarizes the distribution of the six audiovisual patterns across the three scenarios. S1 consisted entirely of P1, Natural landscape, across all 33 retained 1-s intervals (100%). S2 contained four patterns: P2, Green landscape with traffic sounds, was most frequent with 16 intervals (39.02%), followed by P6, Traffic corridor with vegetation, with 13 (31.71%), P5, Open road corridor, with 10 (24.39%), and P1 with two (4.88%). S3 contained three patterns: P4, Built road corridor, accounted for 15 intervals (41.67%), P5 for 12 (33.33%), and P3, Railway infrastructure, for nine (25.00%).

**Table 3:** Distribution of the six audiovisual patterns across the three scenarios.

Pattern/Scenerio	P1	P2	P3	P4	P5	P6	Total
S1	33 (100.00%)	0	0	0	0	0	33
S2	2 (4.88%)	16 (39.02%)	0	0	10 (24.39%)	13 (31.71%)	41
S3	0	0	9 (25.00%)	15 (41.67%)	12 (33.33%)	0	36

Values indicate the number and percentage of retained 1-s intervals assigned to each pattern within the corresponding scenario.

Most patterns were strongly associated with particular scenarios: P1 dominated S1, P2 and P6 occurred only in S2, and P3 and P4 occurred only in S3. Pattern identity could therefore not be separated from scenario and temporal position in the fixed-order stimulus.

Within S2, P2, P5, and P6 each recurred in multiple non-contiguous temporal segments (three, two, and two segments, respectively). In contrast, P3, P4, and P5 in S3 occurred as successive contiguous blocks. This difference supported a restricted exploratory sensitivity analysis within S2 but not an equivalent inferential comparison within S3.

#### 3.3.3 Descriptive facial-expression scores across audiovisual patterns

Facial-expression scores were summarized descriptively across the six audiovisual patterns at the 1-s interval level (Table 4). Because successive intervals were temporally dependent and the identified patterns were strongly associated with particular scenarios and temporal positions, no inferential comparisons were conducted across the six patterns. In addition, because the seven DeepFace scores sum to 100%, differences in individual components represent relative changes within the complete score composition rather than independent changes in emotional intensity.

**Table 4:** Descriptive mean relative facial-expression scores across the six audiovisual patterns.

Pattern	Angry	Disgust	Fear	Happy	Sad	Surprise	Neutral
P1 (*n* = 35)	15.81	0.07	18.69	9.45	14.64	8.92	32.41
P2 (*n* = 16)	16.38	0.07	18.84	8.30	19.01	7.49	29.91
P3 (*n* = 9)	15.42	0.12	18.58	7.94	17.61	7.08	33.26
P4 (*n* = 15)	14.63	0.13	21.77	7.27	16.35	6.82	33.02
P5 (*n* = 22)	14.03	0.17	20.89	7.57	17.51	6.34	33.48
P6 (*n* = 13)	15.33	0.10	17.76	9.19	18.98	8.07	30.58

Values are descriptive mean relative percentage scores. For each retained 1-s interval, scores were first averaged across the 49 participants and were then averaged across the intervals assigned to each audiovisual pattern. *n* indicates the number of retained 1-s intervals assigned to each pattern. The seven DeepFace output components sum to 100% and therefore form a compositional outcome. Because successive intervals were temporally dependent and pattern occurrence was strongly associated with scenario and temporal position, no inferential comparisons were conducted across all six patterns.

The neutral component had the highest mean relative score across all six patterns, ranging from 29.91% in P2 to 33.48% in P5. Among the non-neutral components, sad and fear showed the largest ranges across patterns. P1, Natural landscape, showed the lowest relative sad score (14.64%), whereas the remaining patterns ranged from 16.35 to 19.01%. The relative fear score was descriptively highest in P4, Built road corridor (21.77%), followed by P5, Open road corridor (20.89%), while the remaining four patterns ranged from 17.76 to 18.84%. Angry, happy, and surprise showed comparatively narrower variation across patterns, while disgust remained close to zero throughout.

These values describe differences in the relative composition of DeepFace scores across the audiovisual patterns. Because pattern identity could not be separated from scenario and temporal position, these differences cannot be interpreted as independent effects of audiovisual pattern.

As an exploratory sensitivity analysis within S2, participant-level mean relative scores were compared among P2, P5, and P6 using component-wise Friedman tests. No component showed a statistically significant difference: angry, χ^2^(2) = 2.490, *p* = 0.288; disgust, χ^2^(2) = 2.103, *p* = 0.349; fear, χ^2^(2) = 1.959, *p* = 0.375; happy, χ^2^(2) = 0.875, *p* = 0.646; sad, χ^2^(2) = 2.903, *p* = 0.234; surprise, χ^2^(2) = 0.866, *p* = 0.648; and neutral, χ^2^(2) = 1.830, *p* = 0.401. After Holm adjustment across the seven tests, all remained non-significant (all adjusted *p* = 1.000). These tests were interpreted as exploratory marginal comparisons of relative components within the seven-part composition and were not used to infer general effects across the six patterns.

## 4 Discussion

### 4.1 Scenario-level audiovisual characteristics and facial-expression scores

The three scenarios differed clearly in their overall visual and acoustic characteristics, ranging from the more natural and lower-sound-level conditions of S1 to the stronger transport-related visual and acoustic conditions of S2 and S3. Previous studies have generally associated natural visual and acoustic cues with more favorable affective and restorative evaluations, whereas traffic noise tends to reduce environmental quality and perceived restoration (Annerstedt et al., 2013; Korpilo et al., 2024; Van Renterghem and Lippens, 2024). Such evidence might suggest corresponding differences in affective response. In the present study, however, none of the seven relative facial-expression components differed significantly among scenarios after Holm adjustment. These exploratory component-wise results do not establish equivalence of the overall seven-part composition across scenarios.

This apparent discrepancy may partly reflect the mixed sensory conditions represented by the stimuli. Unlike controlled experiments contrasting relatively homogeneous natural and urban environments or pairing isolated scenes with specific sounds (Carles et al., 1992; Li and Lau, 2020; Lu et al., 2023), the present stimuli retained the coexistence of natural and transport-related information found in an actual UVGS. S2 illustrates this clearly: natural features accounted for 47.15% of the visual composition, whereas built and transport-related features accounted for 52.34%, together with persistent road-traffic sounds. The scenarios therefore represented broad environmental contexts rather than sharply separated natural and transport conditions, and the relevance of individual features may depend on the other visual and acoustic information present.

Participant-level averaging across each complete scenario may also have reduced sensitivity to within-scenario variation. S1 consisted entirely of one audiovisual pattern, whereas S2 and S3 contained several patterns. Averaging facial-expression scores across each full segment therefore combined variation occurring under different audiovisual patterns into a single scenario-level measure.

The absence of statistically supported component-wise differences therefore indicates only that the three broad scenarios did not yield detectable differences in the relative facial-expression components examined here. This result prompted a finer-grained descriptive examination of facial-expression scores across the identified audiovisual patterns.

### 4.2 Audiovisual patterns and variation in facial-expression scores

The pattern-level analysis provided a finer-grained descriptive view of facial-expression scores across the identified audiovisual patterns. However, pattern occurrence was strongly associated with scenario and temporal position, and all scenarios were presented in a fixed order. The observed differences therefore cannot be interpreted as independent pattern effects. In addition, because the seven DeepFace scores form a relative composition summing to 100%, variation in any individual component should be interpreted in relation to the remaining components.

Within these limitations, several descriptive tendencies were observed. P1, Natural landscape, showed the lowest mean relative sad score among the six patterns. This pattern combined vegetation and water with bird vocalizations and flowing water, and this descriptive tendency is broadly compatible with studies associating natural scenes and sounds with more favorable affective and restorative evaluations (Benfield et al., 2010; Jo and Jeon, 2021; Uebel et al., 2021). However, the present design cannot determine whether this tendency reflects the audiovisual pattern itself, the broader scenario context, temporal position, or their combination.

Relative fear scores were descriptively highest in P4, Built road corridor, and P5, Open road corridor, whereas the remaining four patterns showed lower and relatively similar values. Both P4 and P5 combined prominent transport-related sounds with pronounced built and roadway visual features. This tendency is broadly compatible with previous evidence that visual context may influence the appraisal of environmental sounds (Viollon et al., 2002), although the present design does not allow visual, scenario, and temporal effects to be separated.

Other components showed comparatively narrower descriptive variation across patterns. In particular, the surprise component ranged from 6.34 to 8.92%; because surprise has no fixed positive or negative valence, this variation should not be interpreted as indicating a more or less favorable response (Reisenzein et al., 2019).

The restricted sensitivity analysis within S2 found no statistically significant component-wise differences among P2, P5, and P6 after Holm adjustment. These tests were treated only as exploratory marginal comparisons within the seven-part composition and provided no basis for broader inference across the six patterns.

Overall, the pattern-level findings are best regarded as exploratory and hypothesis-generating. They describe recurring audiovisual patterns and the facial-expression responses observed alongside them but do not establish independent pattern effects. Future studies should repeat comparable patterns across scenarios and presentation orders to better separate pattern, scenario, and temporal effects.

### 4.3 Implications for evaluating audiovisual experience in under-viaduct green spaces

Taken together, the findings suggest that audiovisual experience in UVGSs cannot be adequately characterized by isolated environmental indicators. Vegetation proportion describes visual greenness, while $L_{\mathrm{Aeq}}$ characterizes overall sound level, but neither captures how natural and transport-related information co-occurs within the same setting. The pattern analysis further showed that a single scenario may contain several recurring audiovisual patterns. Evaluation should therefore consider both overall environmental conditions and the specific combinations of visual features and sound sources occurring within them.

This perspective is relevant to the restorative potential of UVGSs because natural features such as vegetation and water are experienced together with roads, vehicles, viaduct structures, and traffic sounds. Their potential contribution to affective and restorative experience may therefore depend on the broader multisensory context rather than on visual greenness alone (Annerstedt et al., 2013).

For practical evaluation, assessments of UVGSs should complement conventional indicators of vegetation and sound level with information on visual context, sound-source composition, and their co-occurrence. This approach is consistent with broader calls for transparent and evidence-based sustainability assessment, particularly where environmental claims should be supported by verifiable evidence (Raman et al., 2025). Identifying recurring audiovisual patterns, together with the descriptive facial-expression scores observed alongside them, can support a more differentiated description of environmental experience and help characterize how natural features are situated within the surrounding sensory context. Such evidence can inform regeneration and management without assuming that increasing greenery or reducing overall sound level alone will produce equivalent experiential outcomes.

### 4.4 Limitations and future research

Several limitations should be considered. First, the study examined a single UVGS in Shanghai using a relatively homogeneous undergraduate sample. The six audiovisual patterns should therefore be regarded as patterns identified in the present dataset rather than as a general typology of UVGSs. Their occurrence and associated facial-expression responses may vary across sites, traffic conditions, and user groups. Future studies should include multiple sites and more diverse participants.

Second, recorded audiovisual stimuli enabled standardized presentation but simplified in-situ experience. The fixed presentation order also prevented separation of order, habituation, and fatigue effects. Future studies could use counterbalanced designs and incorporate field-based or immersive approaches. In addition, field-measured $L_{\mathrm{Aeq}}$ values characterized recording conditions rather than participants’ auditory exposure because headphone playback was not calibrated at the ear.

Third, DeepFace-derived scores were used to characterize observable facial-expression responses rather than subjective emotional states. Because the seven scores sum to 100%, they form a compositional outcome; category-specific Friedman tests were therefore treated only as exploratory marginal comparisons with Holm adjustment. Individual categories, particularly those without fixed valence such as surprise (Baudouin et al., 2025), should be interpreted cautiously. Future studies could combine facial-expression analysis with self-report, physiological, or behavioral measures and apply multivariate or compositional-data methods.

Finally, the pattern analysis was based on successive 1-s intervals from a continuous fixed-order stimulus. The 1-s interval served as a common sampling and synchronization unit rather than a discrete psychological response window; temporal lag, accumulation, and carry-over may occur. Adjacent intervals were temporally dependent, and pattern occurrence was strongly associated with scenario and temporal position. The present design therefore cannot separate independent pattern effects from scenario and temporal effects, and pattern-level results were interpreted descriptively. Future studies should repeat comparable patterns across scenarios and presentation orders, examine alternative temporal windows and lagged relationships, and use methods that explicitly account for participant-level and temporal dependence.

## 5 Conclusion

This study characterized the audiovisual conditions of an under-viaduct green space and the facial-expression responses observed during exposure. The three scenarios differed clearly in visual composition, overall sound level, and sound-source composition, but exploratory component-wise analyses found no statistically significant differences in any of the seven relative facial-expression scores after Holm adjustment. Six recurring audiovisual patterns were identified, with descriptive variation in the relative DeepFace scores observed across them. These pattern-level differences should be regarded as exploratory tendencies because pattern occurrence was closely associated with scenario and temporal position and cannot be interpreted as independent pattern effects or as direct evidence of subjective emotional states.

The findings highlight the importance of considering natural and transport-related sensory information jointly when evaluating UVGSs. Rather than relying only on vegetation proportion or overall sound level ( $L_{\mathrm{Aeq}}$ ), evaluation should also consider visual context, sound-source composition, and recurring audiovisual patterns within the site. This integrated perspective provides a more differentiated basis for evaluating audiovisual experience in green spaces embedded within transport infrastructure and can inform their future regeneration and management.

## Data Availability

The original contributions presented in the study are included in the article/supplementary material, further inquiries can be directed to the corresponding author.
